# Evaluation of nutraceutical properties of *Leucaena leucocephala* leaf pellets fed to goat kids infected with *Haemonchus contortus*

**DOI:** 10.1186/s12917-020-02471-8

**Published:** 2020-08-10

**Authors:** Carine Marie-Magdeleine, Steve Ceriac, Dingamgoto Jesse Barde, Nathalie Minatchy, Fred Periacarpin, Frederic Pommier, Brigitte Calif, Lucien Philibert, Jean-Christophe Bambou, Harry Archimède

**Affiliations:** 1 INRAE, UR143, Unité de Recherches Zootechniques, French West Indies, 97170 Petit-Bourg, Guadeloupe; 2 INRA UE1284, Plateforme Tropicale d’Expérimentation sur l’Animal, French West Indies, 97170 Petit-Bourg, Guadeloupe

**Keywords:** goat kid, *Leucaena leucocephala*, nutraceutical, gastrointestinal parasite, feed, anthelmintic, growth

## Abstract

**Background:**

*Leucaena leucocephala*, as a shrub legume rich in condensed tannins, is a candidate for the integrated control of gastrointestinal parasitic pathogen nematodes. Here, we investigated the anthelmintic potential of the nutraceutical *L. leucocephala*, transformed into pellets, against *Haemonchus contortus*.

**Results:**

Creole goat kids were fed an iso-nitrogenous diet of *Dichantium* hay with alfalfa pellets or *Leucaena* pellets or an alfalfa–leucaena mixture in varying ratios. The artificial infection of kids with *H. contortus* led to infection levels comparable to those observed in the farm environment on the basis of egg excretion. The anthelmintic activity of *Leucaena*, compared to alfalfa, was demonstrated by its potential to reduce egg excretions (1524 vs. 3651 eggs/g) and the larval development of excreted eggs (3.5 vs. 24%). This anthelmintic potential was reported when the ratio of *Leucaena* incorporation in the diet was high (50% DM intake). The voluntary dry matter intake (79.3 vs. 77.0 g Large Weigth^0.75^), the total tract dry matter digestion (0.585 vs. 0.620), and the growth (57.1 vs. 71.3 g/d) of kids fed *Leucaena* compared to alfalfa indicate that *Leucaena* has a high feed value. The *Leucaena*, even at the highest intakes in the diets, has not shown any signs of poisoning in kids.

**Conclusions:**

*Leucaena* fulfilled the conditions to be a good nutraceutical, and pelleting is a good method for its use.

## Background

Gastrointestinal parasitic nematodes (GINs) in small ruminants are a major constraint on animal health and well-being and the economic profitability of farms, and the magnitude of the problem is accentuated in the humid tropics [1]. Today, the main method used to control GINs is still the use of synthetic chemical anthelmintics; however, the resistance of parasites to chemical molecules weakens this control strategy [2–4]. There is thus an urgent need to develop integrated control methods [5, 6] combining the genetic selection of resistant and resilient animals, pasture management, adapted nutrition, and anthelmintic plants. As part of this integrated control, research into the use of some nutraceutical plants as feed [7, 8] rich in condensed tannins (CTs) and protein roughage is ongoing. The CTs and protein overnutrition have direct and indirect effects on the development and effects of GINs. The beneficial effect of CTs against worms can be offset by their depressive effect on the feed value of the forage, as demonstrated by a reduction in intake and digestion. It is therefore advisable to evaluate the nutraceutical according to at least these two criteria: feed and anthelmintic value. The dosage (amount and rate of intake) of the nutraceutical is also an open question. The value of the nutraceutical should be assessed according to its efficacy against GINs that have already acquired resistance to synthetic chemical anthelmintics and its potential to generate new resistance in GINs. Finally, technological innovations, such as the form of presentation of the nutraceutical, could allow the use of a nutraceutical more appropriate to the scale of the farm’ [8–10]. In small farmed ruminants, goats are the most sensitive species to GINs. Within this species, kids are the most sensitive animals. Consequently, kids are used as model animals to study this pathology. The main objective of this study was to evaluate the feed and anthelmintic potential of the nutraceutical *Leucaena leucocephala*, a shrub legume rich in condensed tannins, transformed into pellets. We evaluated the effect of several doses of pellets in the diet and the efficiency of *L. leucocephala* against *Haemonchus contortus*. Some preliminary results of this study were reported at the 10th International Symposium on the Nutrition of Herbivores [11].

## Results

Results beyond 29 post-infection only concern 4 animals per lot due to slaughter.

The intake and total tract digestion values are reported in Table 1. Whatever the mode of expression (g/d, g/kg LW^0.75^, g/kg LW), the total dry matter (DM) intakes were similar for non-infected kids fed supplements composed of 100% alfalfa or *Leucaena*. Comparison of these last diets revealed that the DM intake was lower or tended to be lower for non-infected kids than for infected kids. The DM total tract digestion tended to be lower (P = 0.07) in non-infected kids fed supplements composed of 100% *Leucaena* than those fed with 100% alfalfa. No difference in the DM total tract digestion was reported for non-infected kids and infected kids fed diets with supplements composed of 100% alfalfa. However, a lower DM total tract digestion was reported for infected kids than non-infected kids and kids fed diets with supplements composed of 100% *Leucaena*. The digestible crude protein (CP) intakes were similar for non-infected kids fed supplements composed of 100% alfalfa or *Leucaena*.

**Table 1:** Intake of uninfected (U) or infected (I, with *H. contortus*) Creole kids eating: *Dichanthium spp.* hay + alfalfa pellet (A); *Dichanthium spp.* hay + *L. leucocephala* pellet (L); *Dichanthium spp.* hay + (25% *L. leucocephala* pellet + 75% alfalfa pellet; 25 L); *Dichanthium spp.* hay + (50% *L. leucocephala* pellet + 50% alfalfa pellet; 50 L)

				Treatment					*P-*value	
Item	U-A	I-25 L	I-50 L	I-A	U-L	I-L	SE	Treatment	Diet	Infection
Dry Matter g/d /Live Weight ^0.75^	77.0ab	82.0 a	78.9a	73.6b	79.3a	70.8b	2.27	0.0024	0.0037	0.0289
Dry Matter g/Live Weight	41.5ab	44.0a	42.9b	39.9a	42.4a	39.2b	1.18	0.0370	0.0286	0.1269
Digestible Dry Matter/Live Weight ^0.75^	48.2a	51.7a	49.5a	45.8a	46.8a	39.6b	1.99	< 0.0001	0.009	0.0440
Dry Matter, g/d	497.4b	535.0b	466.5a	497.2ab	521.8b	414.7c	25.05	0.0079	0.0119	0.0189
Organic Matter, g/d	455.1b	489.1b	425.5a	454.5ab	481.0b	381.8c	22.89	0.0098	0.0134	0.0158
Crude Protein, g/d	83.7c	108.2b	84.1c	90.8a	102.9b	86.1ac	5.34	0.0078	0.0012	0.1690
Digestible Crude Protein g/Live Weight ^0.75^	8.7a	9.9a	8.4b	8.0a	7.5a	6.6c	0.56	< 0.0001	0.0015	0.3743
Neutral Detergent Fibre, g/d	298.7a	292.9a	295.0a	269.4a	283.9a	217.5b	13.76	< 0.0001	0.0010	0.0039
Acid Detergent Fibre, g/d	173.4a	157.0a	168.7a	159.6a	144.9a	110.9b	7.68	< 0.0001	< 0.0001	0.0097
Consumption index, g feed/g Daily Growth	7.0b	6.3ab	6.9a	6.5ab	9.2c	7.2b	0.50	0.0152	0.0396	0.0366

a, b, c: Means lacking a common letter differ (*p* < 0.05)

No significant effect of parasitism on total tract digestion (ttd) of CP, neutral detergent fibre (NDF) or acid detergent fibre (ADF) was found for diets supplemented with 100% alfalfa (Table 2). The CPttd of diets supplement with 100% *Leucaena* was lower for infected kids than for non-infected kids, whereas no difference was observed for NDF and ADF. The average daily gain (ADG) values are reported in Table 3. Overall, the ADG was lower with the *Leucaena* diet than the alfalfa and alfalfa–*Leucaena* diets (Table 3). No difference was recorded between these last two diets. Within the same diet, no difference in growth was found between infected and non-infected kids.

**Table 2:** Total tract digestibility of uninfected (U) or infected (I, with *H. contortus*) Creole kids eating: *Dichanthium spp.* hay + alfalfa pellet (A); *Dichanthium spp.* hay + *L. leucocephala* pellet (L); *Dichanthium spp.* hay + (25% *L. leucocephala* pellet + 75% alfalfa pellet; 25 L); *Dichanthium spp.* hay + (50% *L. leucocephala* pellet + 50% alfalfa pellet; 50 L)

				Treatment					*P-*value	
Item	U-A	I-25 L	I-50 L	I-A	U-L	I-L	SE	Treatment	Diet	Infection
Dry Matter	0.620a	0.625a	0.624a	0.617a	0.585ac	0.548b	0.0143	< 0.0001	0.0016	0.2363
Organic Matter	0.585a	0.590a	0.586a	0.587a	0.550c	0.509b	0.0157	< 0.0001	0.0028	0.2143
Crude Protein	0.648a	0.651a	0.649a	0.675a	0.569a	0.516b	0.0166	< 0.0001	< 0.0001	0.5166
Neutral Detergent Fibre	0.640a	0.595a	0.631a	0.605a	0.558b	0.494b	0.0231	< 0.0001	< 0.0001	0.0565
Acid Detergent Fibre	0.614a	0.520a	0.592ab	0.582b	0.442c	0.352c	0.0333	0.01010	< 0.0001	0.1029
Average Daily Gain (ADG) (g/d)	71.3a	75.9a	90.0b	74.6a	57.1c	59.9c	4.49	0.0669	0.0002	0.5147

a, b, c: Means lacking a common letter differ (*p* < 0.05)

**Table 3:** Partition of nitrogen intake and growth of uninfected (U) or infected (I, with *H. contortus*) Creole kids eating: *Dichanthium spp.* hay + alfalfa pellet (A); *Dichanthium spp.* hay + *L. leucocephala* pellet (L); *Dichanthium spp.* hay + (25% *L. leucocephala* pellet + 75% alfalfa pellet; 25 L); *Dichanthium spp.* hay + (50% *L. leucocephala* pellet + 50% alfalfa pellet; 50 L)

				Treatment					*P-*value	
Items	U-A	I-25 L	I-50 L	I-A	U-L	I-L	SE	Treatment	Diet	Infection
N intake g/d	13.4a	17.0a	15.0a	13.5a	16.5a	12.7b	0.88	0.1217	0.0012	0.1690
N in faeces g/d	4.5c	6.0c	5.1ab	4.3a	7.1b	6.3ab	0.33	0.0015	< 0.0001	0.3943
N in urine g/d	5.2c	7.1c	6.3a	5.9a	3.2b	4.7b	0.44	< 0.0001	0.0013	0.0174
N retained	3.8a	3.9a	3.5a	3.6a	6.2b	1.8a	0.79	0.0669	0.7324	0.0234
Average Daily Gain predicted (g/d)	114.9a	110.7a	106.5a	109.3a	189.3c	54.7b	24.83	0.0669	0.7112	0.0114
Average Daily Gain (ADG) (g/d)	58.0a	90.0b	74.5b	66.0a	57.1c	59.9c	4.49	0.0669	0.0017	0.5498

a, b, c: Means lacking a common letter differ (*p* < 0.05)

Overall, the feed indices tended to be higher for the *Leucaena* diet than the alfalfa and alfalfa–*Leucaena* diets; however, the difference was only significant for the uninfected kids fed *Leucaena*. Within the same diet, the differences were significant only with *Leucaena*, where values were significantly higher with non-infected kids.

The partition of nitrogen flows is presented in Table 3. Overall, nitrogen intakes were lower with *Leucaena* than with the other diets. When nitrogen intake is taken into account as a covariate in the statistical model, faecal and urinary nitrogen excretion is similar to that shown in Table 3. The faecal nitrogen excretions were lower with the alfalfa diets compared to the *Leucaena* diet. No difference was observed between infected and non-infected kids fed the same diet. When nitrogen intake is taken into account as a covariate in the statistical model, urinary excretion is lower with the *Leucaena* diet than the alfalfa diet. *Leucaena* diet leads to lower urinary nitrogen excretions than the alfalfa–*Leucaena* diet. No difference in urinary excretion was observed between infected and non-infected kids fed the same diet. Only non-infected kids fed the *Leucaena* diet had significantly higher values of retained nitrogen compared to the other treatments. The predicted ADG values were higher than those measured and the tendencies observed were similar to those reported for retained nitrogen.

**Fig. 1:**
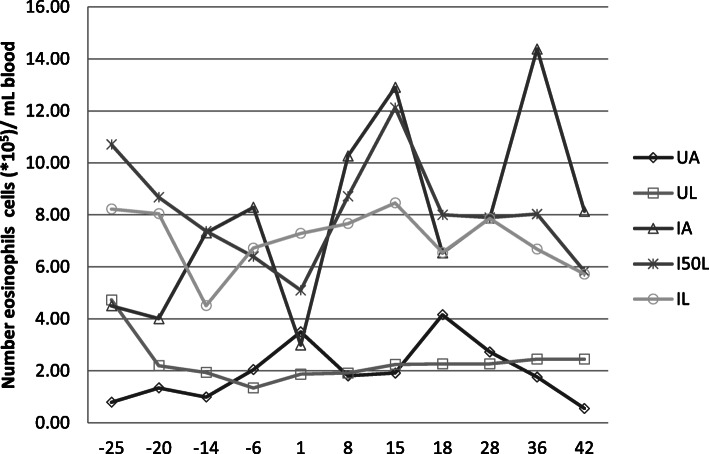
Evaluation of eosinophils from uninfected Creole kids and infected Creole kids eating various diets: kids infected with *Haemonchus contortus* and fed hay + 300 g (62% alfalfa + 38% soybean) (∆); kids infected with *Haemonchus contortus* and fed hay + 300 g (47% alfalfa + 25% *Leucaena* + 28% soybean) (x); kids infected with *Haemonchus contortus* and fed hay + 300 g (30% alfalfa + 50% *Leucaena* + 20% soybean) (*): kids infected *Haemonchus contortus* and fed hay + 300 g *Leucaena* (○); uninfected kids fed hay + 300 g (62% alfalfa + 38% soybean) (◊); uninfected kids fed hay + 300 g *Leucaena* (□). Results beyond 29 post-infection only concern 4 animals per lot due to slaughter

The weekly evaluations of eosinophils are summarised in Fig. 1. There was no difference between the two control diets whichever time point was considered. The eosinophil counts post infections were higher with infected kids eating Alfalfa (IA) than with uninfected kids eating Alfalfa (UA); however, differences were only significant in weeks 2 and 5. The eosinophil counts post infection were higher with infected kids eating *L. leucocephala* (IL) than with uninfected kids eating *L. leucocephala* (UL). No significant difference was observed between infected kids regardless of their diet.

**Fig. 2:**
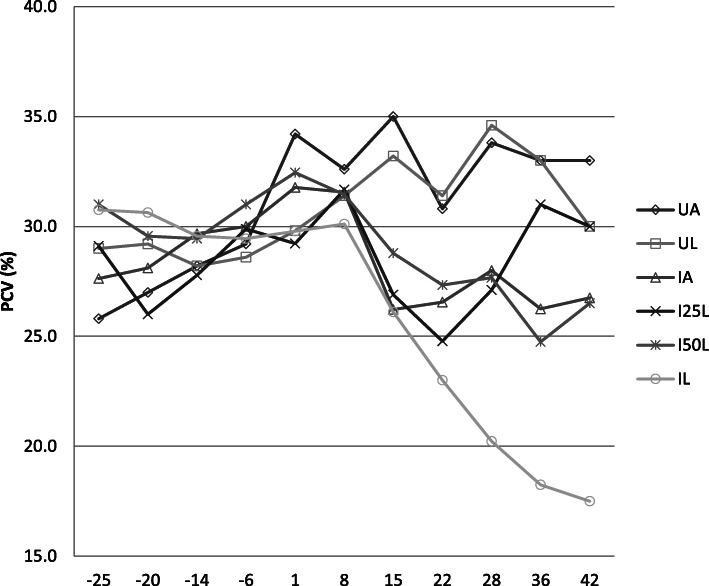
Evaluation of PCV in uninfected Creole kids and infected Creole eating various diets; kids infected with *Haemonchus contortus* and fed hay + 300 g (62% alfalfa + 38% soybean) (∆); kids infected with *Haemonchus contortus* and fed hay + 300 g (47% alfalfa + 25% *Leucaena* + 28% soybean) (x); kids infected with *Haemonchus contortus* and fed hay + 300 g (30% alfalfa + 50% *Leucaena* + 20% soybean) (*): kids infected *Haemonchus contortus* and fed hay + 300 g *Leucaena* (○); uninfected kids fed hay + 300 g (62% alfalfa + 38% soybean) (◊); uninfected kids fed hay + 300 g *Leucaena* (□). Results beyond 29 post-infection only concern 4 animals per lot due to slaughter

The weekly evaluations of blood packed cell volume (PCV) are summarised in Fig. 2. The PCVs did not vary with time for the two control diets. Furthermore, there was no difference between the two control diets whichever time point is considered. The PCV values post infection were lower with IA than with UA; however, the differences were only significant in weeks 2, 3, and 4. No significant difference was observed between infected kids regardless of their diet, except for the comparison of IA vs. IL, for which the values were lower with *Leucaena* in weeks 3, 5, and 6 post infection.

**Fig. 3:**
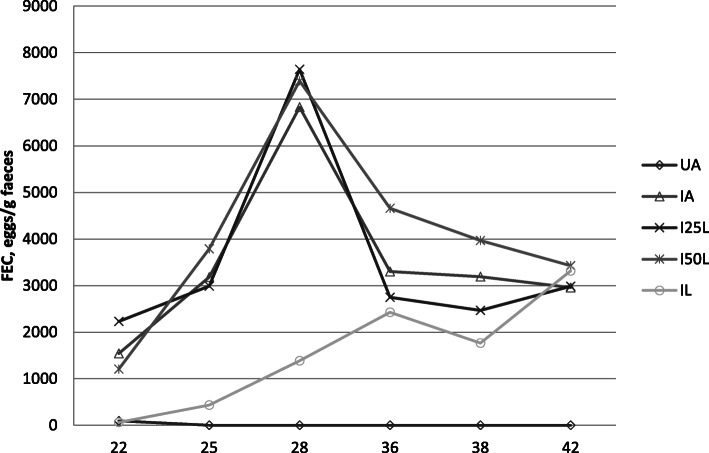
Evaluation of FECs from uninfected Creole kids and infected Creole kids eating various diets: kids infected with *Haemonchus contortus* and fed hay + 300 g (62% alfalfa + 38% soybean) (∆); kids infected with *Haemonchus contortus* and fed hay + 300 g (47% alfalfa + 25% *Leucaena* + 28% soybean) (x); kids infected with *Haemonchus contortus* and fed hay + 300 g (30% alfalfa + 50% *Leucaena* + 20% soybean) (*): kids infected *Haemonchus contortus* and fed hay + 300 g *Leucaena* (○); uninfected kids fed hay + 300 g (62% alfalfa + 38% soybean) (◊); uninfected kids fed hay + 300 g *Leucaena* (□). Results beyond 29 post-infection only concern 4 animals per lot due to slaughter

The weekly evaluation of faecal egg count (FEC) are summarised in Fig. 3. As expected, the FEC was zero for the uninfected kids. The FECs varied with time, as expected. The FEC values post infection were significantly lower with IL than with IA from weeks 3 to 6. By week 7, the difference became insignificant for IL vs. IA.

No effect of diet was found on the total number of male and female worms (Table 4). The number of immature male and female worms was higher with *Leucaena* diets. The sex ratio of mature male/ mature female worms was lower with Alfalfa diet. The number of eggs in utero was lower with the *Leucaena* diets than with the other diets. The same tendency was observed for worm lengths, with the lowest values reported for *Leucaena* diets. Faecal eggs development decreased with the amount of *leucaena*.

**Table 4:** Faecal eggs development and worm characteristics in the abomasum of uninfected (U) or infected (I, with *H. contortus*) Creole kids eating: *Dichanthium spp.* hay + alfalfa pellet (A); *Dichanthium spp.* hay + *L. leucocephala* pellet (L); *Dichanthium spp.* hay + (25% *L. leucocephala* pellet + 75% alfalfa pellet; 25 L); *Dichanthium spp.* hay + (50% *L. leucocephala* pellet + 50% alfalfa pellet; 50 L)

			Treatment			*P-*value		
Item	I-A	I-25 L	I-50 L	I-L	SE	Treatment	*Leucaena*	
Total worms	773.8	982.0	705.8	897.6	187.44	0.750	0.506	
Male (%)	43.3	51.3	48.5	46.2	2.32	0.1693	0.1252	
Female (%)	56.7	55.8	51.3	53.8	2.24	0.4326	0.3324	
Immature worms (%)	1.7a	2.4a	3.0a	9.7b	2.20	0.0265	0.0111	
Immature male worms (%)	0.9a	1.6a	1.0a	5.6b	1.21	0.0140	0.0080	
Immature female worms (%)	2.2a	2.8a	4.4a	13.0b	3.03	0.0315	0.0130	
Sex ratio mature male female (%)	79.4	93.7	98.1	94.0	6.34	0.0485	0.0770	
Female worm length, cm	1.929ab	1.951a	1.921ab	1.838ab	0.0444	0.0128	0.0261	
Eggs in utero	209.3ab	242.1a	247.3a	102.2bc	30.76	0.0010	0.0032	
Faecal eggs development (%)	24.0a	14.0b	12.7b	3.5c	3.50	0.0009	< 0.0001	

a, b, c: Means lacking a common letter differ (*p* < 0.05)

## Discussion

The chemical composition of *Leucaena* was agreement with previous results reported [12]. The intake and digestibility decreases observed for sick animals compared to healthy animals. This is a relatively frequent, though unsystematic, as reported in the literature [13–15]. A large reduction in intake 3 weeks post infection is reported [14], which is quite comparable to our observations. However, variable impacts (lag time post infection and the magnitude and duration of intake reduction) of gastrointestinal parasitism are observed, probably related to the animal’s history, the infecting strain, the extent of the infection, and the feed characteristics [14]. The depressive effect of gastrointestinal parasites on total digestibility has also been reported in a literature review [15]. A meta-analysis reported that for a one-unit increase in log-transformed FEC, the DM intake and organic matter digestibility decreased by 8.68 g/kg LW^0.75^ and 1.21% respectively, for sheep and goats [15].

The reduction in the PCV from 1 week after infection is a classical result that elicits the haematophagous activity of *H. contortus* [16]. A meta-analysis reported that for a one-unit increase in log-transformed FEC, PCV decreased by 62.8% and 23.4% for sheep and goats, respectively [15]. Thus, gastrointestinal parasitism increases the nutrient requirement (energy, proteins, and minerals) to compensate for some endogenous losses (blood, sloughed cells, etc.) [17]. Feeds rich in protein increase the resilience of animals, counterbalancing the haematophagous action of *H. contortus*, as observed with the alfalfa and mixed alfalfa–*Leucaena* diets. The lowest PCVs recorded with *Leucaena*, comparing uninfected to infected kids, reveals a lower availability of nutrients. This result is in agreement with lowest digestible DM and digestible CP intake being recorded with infected kids fed *L. leucocephala*.

To our knowledge, the literature does not mention in vivo trials demonstrating the nutraceutical properties of *Leucaena* leaves, although anthelmintic properties of the seeds have been shown. However, the results concerning health indicators show an expected anthelmintic activity of this forage, probably due to the presence of the CTs. Indeed, the anthelmintic properties of CTs have been widely documented in the literature [8, 18–20].

Feeding pelleted *L. leucocephala* was effective in reducing GIN infection in kids, although drying and heating have a depressive effect on the CT content of forages. Several authors [9, 21, 22] have reported similar results with pellets of *Espedeza cuneate*. In our study, the method used to produce the pellets was sun drying followed by heating during pelleting at a temperature below 70 °C for less than 5 min. We hypothesise that this method did not destroy the anthelmintic properties of the CTs. This result is particularly interesting, since the CTs of *Leucaena* would be more sensitive to drying and heating than those of other species [23].

The anthelmintic activity was only found with high levels of incorporation of *L. leucocephala* in the diet. Our study indicates that the anthelmintic effect appears only with the highest levels (60% DMI) of *L. leucocephala*, and thus CTs (4.5% DMI) in the diet. This result is similar to those presented in the literature [19], indicating that approximately 30 to 40 g CT/kg DM is useful for anthelmintic activity. It is likely that this range of values varies according to the characteristics of the CTs. The anthelmintic activity of CTs is more attributable to a reduction in the fertility of female worms rather than to the survival rate of larvae [24]. This experiment demonstrates the effects of tannins at the different stages of the parasite cycle, as reported by Hoste [7]. The reduction in the number of eggs in female worms together with a reduction in their length with the *Leucaena* diet could illustrate a decrease in their fecundity. The reduction of daily eggs excreted with *Leucaena* seems to validate this hypothesis because the number of female worms is similar between diets. The increase in the proportion of immature female worms with *Leucaena* relative to the other treatments might also explain the low female fertility. In addition, the reduction in the Faecal eggs development into larvae in relation to CTs demonstrated in this study is consistent with previous results [7]. This could be due to a direct or indirect effect on egg physiology. A reduced establishment of infective third-stage larvae with tannins is also reported by Brunet [25].

## Conclusions

*L. leucocephala* pellets have the two main properties of a nutraceutical: feed and anthelmintic. As a feed, *L. leucocephala* is high in protein, enabling Creole goats to reach approximately 80% of their growth potential. As an anthelmintic, *L. leucocephala* reduces the faecal eggs development of eggs into larvae, therefore limiting environmental contamination and reducing the fertility of female worms.

## Methods

The experimental design was designed to evaluate the anthelmintic activity of increasing doses of *L. leucocephala* pellets on *Haemonchus contortus*. To avoid possible bias, the assessors were blinded to any stages of the methodological process.

## Feed

The diets consisted of hay, alfalfa pellets, *Leucaena* leaf pellets, and soybean meal. The composition of the ingredients is shown in Table 5. The hay used was mainly composed of 60-day-old *Dichanthium spp.* from a fertilised and irrigated natural savannah grass. *L. leucocephala* was a mixture from collections on 9–12-month-old fallow, unfertilised farmlands. The soils were diversified: deep ferralitic soils, allophane and halloysite soils, and vertisols. The young stems and leaves, less than 6 months old, were harvested and then sun dried for 2 days. The leaves with petioles were separated from the stem, ground (through a 3-mm screen), and granulated with a GR150E system (Oliotechnology, Burgun, Wissembourg, France). The average size of the pellets was 12 mm long by 3 mm in diameter. Alfalfa pellets were sourced commercially.

**Table 5:** Chemical composition of the ingredients of the diets

	Chemical compound (g/100 g)					
Ingredients	OM	NDF	ADF	ADL	CP	CT
*Dichanthium* spp. hay	93.4	76.1	39.3	5.0	8.3	.
Alfalfa pellet	89.6	43.0	30.6	7.8	15.8	.
*L. leucocephala* pellet	91.3	38.7	25.2	16.7	26.6	7.5
Soya meal	93.6	12.2	7.3	0.7	45.3	.

*OM * Organic Matter, *NDF * Neutral detergent fibre, *ADF *Acid detergent fibre, *ADL *Acid detergent lignin, *CP *Crude protein, *CT *Total condensed tannins

The four diets were formulated to be iso-nitrogenous. Due to the higher nitrogen content of *Leucaena* than alfalfa, soybean meal was used to balance the nitrogen in the diet. The diet compositions (% raw material) were:

Diet A, Hay *ad libitum* + 300 g (62% alfalfa + 38% soybean).

Diet 25 L, Hay *ad libitum* + 300 g (47% alfalfa + 25% *Leucaena* + 28% soybean).

Diet 50 L, Hay *ad libitum* + 300 g (30% alfalfa + 50% *Leucaena* + 20% soybean).

Diet L, Hay *ad libitum* + 300 g *Leucaena*.

The animals were fed every morning at 8 a.m. with the distribution of pellets and then hay 30 min later. Water was available, *ad libitum*, with automatic distributors.

### Animals and experimental design

Forty-six goat male kids (4 months old, on average 11 ± 1.7 kg at the beginning of the experiment), coming from the farm of the INRA experimental unit (PTEA, Plateforme Tropicale d’Expérimentation sur l’Animal) were used during the 3-month trial. Animal husbandry complies with French legislation. The protocol (APAFIS#5527-2016050608133139v2) has been validated by the Ministry of National Education, Higher Education and Research under the advice of the Ethics Committee for Animal Experiments N°069. The planning of the experiment was: 2 weeks of diet adaptation, 4 weeks of measurements before artificial infection of the required number of the kids, 6 weeks of post-infection measurements, including slaughter of 20 kids 4 weeks post-infection. To ensure the animals started the experiment worm-free, the kids were all previously orally administered praziquantel (Cestocure® Bayer) (3.75 mg/kg body weight (BW)) and ivermectin (Oramec, Merial, Lyon, France), 0.3 mg/kg BW. The FEC levels were checked after treatment. The kids were experimentally infected, 6 weeks after the beginning of the experiment, with a single oral dose of 500 *H. contortus* third-stage infective larvae (L3)/kg live weight. This dose allows to reach a level of infection, measured by the concentration of eggs in the faeces, similar to a high infection under the conditions of grazing farming. The kids were allocated to six lots depending on the diet fed and the parasitic status of the animals (infected vs. uninfected). The six lots were:

Lot UA, five uninfected kids fed Diet A.

Lot UL, five uninfected kids fed Diet L.

Lot IA, nine kids infected with *Haemonchus contortus* and fed Diet A.

Lot IL25, nine kids infected with *Haemonchus contortus* and fed Diet L25.

Lot IL50, nine kids infected with *Haemonchus contortus* and fed Diet L50.

Lot IL, nine kids infected with *Haemonchus contortus* and fed Diet L.

The kids were assigned at random and indifferent to the control or experimental lots which were balanced on the basis of the weight of the kids and their growth between 30 and 90 days after birth. We have excluded extreme kids, too heavy or too light, from an initial population of sixty kids. The size of the lots was based on current practices in this type of experimentation and reported in the literature [15]. Some of the kids were placed in individual boxes (1.5 × 1.5 m size) on the ground (4/lot), except for the uninfected control lot. The other infected kids (5/lot) and the uninfected kids (5/lot) were in metabolic cages (1.5 × 0.5 m size) for the collection of urine (5 consecutive days every 15 days). All animals were equipped with faecal bags for 5 consecutive days every 15 days.

### Measurements and calculations

All measurements were carried out individually on the kids. These measures cover both indicators of kids’ responses to feed and indicators of their health status. The kids were individually weighed at the beginning of the experiment and then fortnightly until the end of the trial (90 days). Daily live weight gain (LWG, g/day) was estimated fortnightly from final minus initial body weight. Feed intake was recorded individually for each kid, from Monday to Friday throughout the experiment, by difference between amounts of feeds offered and refused. The dry matter intake of each ingredient, total intake and its components (organic matter (OM), neutral detergent fibre (NDF), acid detergent fibre (ADF), and crude protein (CP)) were expressed on a daily and metabolic weight basis. The feed efficiency (FE = daily live weight gain/daily feed intake) was estimated for each treatment. The kids were fitted with faecal bags in order to collect the total daily faeces to study the digestibility of the diet. Samples representing 10% of the daily excretion of faeces and urine were taken from each kid to have a representative sample for chemical analysis. Daily samples of faeces were used for DM determination and for chemical analyses. Four measurement periods of total tract digestibility (each one lasting 5 days) were carried out during the experiment: two before and two after the artificial infection. The total tract digestibility was estimated as: (feed intake – faeces excreted)/feed intake.

The blood was sampled weekly from each kid by the jugular vein puncture method using EDTA-coated tubes (Becton Dickinson, Plymouth, UK). A method described by Dawkins [26] was used to estimate the number of circulating eosinophils. Malassez cell counter were used to count the eosinophils. Capillary microhaematocrits (centrifuged for 5 min at 12,000 rpm) were used to estimate the Packed Cell Volume (PCV).

Synchronously with blood sampling, faeces were collected to determine the FEC using the McMaster method based on the microscopic egg counting of an aliquot of suspension from a known volume of faeces. Ten grams of faeces have been previously dissolved in 50 ml of a saturated solution of sodium chloride which causes the eggs to float. Three weeks after the artificial infection, faecal samples from each kid were cultured for 8 days at room temperature following the protocol described by Roberts and O’Sullivan [27] to determine the development of faecal eggs. Three samples per day and per kid were used. Five replicates were performed per kid and per week.

Twenty-nine days after the infection, five kids from each infected lot were slaughtered for worm recovery and numeration. The kids were humanely slaughtered using a captive bolt pistol which hits them on the head producing immediate unconsciousness following by exsanguination. The abomasum was isolated from the animal less than 10 min after slaughter and processed immediately. It was cut open over a tray to catch the contents, which were then placed into a flask. The abomasum wall was washed thoroughly under a stream of tap water and the washings were placed into another flask (washing 1). The abomasal mucosa (after being washed) was retained in a third flask for further processing. The abomasal mucosa was immersed in warm tap water for 4–5 h (37 °C) to recover inhibited or non-inhibited larvae and the remaining juvenile and adult worms (washing 2). Up to 2 L of water were added to the contents during each washing. The contents, mixed with water, were sieved through a 125 mm wire-mesh screen and the washings sieved through a 32 mm wire-mesh screen. Each subsample (corresponding to the contents and washings 1 and 2 for each kid) was mixed manually and three aliquots (100 mL) of each subsample were taken to ensure homogeneous sampling. One 100 mL subsample (i.e., approximately 10% of the total washing volume) was taken per animal. Formalin preservative (35% ethanol, 2.5% formol, qsp 1000 mL distilled water) was added to the subsamples (5%, v/v), which were stored at 4 °C until counting. The worms were identified and counted in each subsample under a binocular magnifier glass (magnification 6.3–50×), separating the males from the females, and the total worm count was determined. The lengths of 10 female worms per kid were measured using a calliper. In utero egg counts were performed on these 10 female worms as described by Kloosterman et al. [28]. The fertility of female worms was estimated by calculating the ratio between the daily excretion of eggs (averaged over the 5 days prior to slaughter) and the number of worms counted in the abomasum.

The liver, kidney, and abomasum wall were observed to identify any necrotic damage and gastritis as indicators of the eventual toxicity of the diet.

### Laboratory analysis

The DM content of diet ingredients, refusals, and faeces was determined by drying at 60 °C in a forced draught oven to constant weight. These samples were used for laboratory chemical analysis after grinding through a 1-mm screen (Reich hammer mill, Haan, Germany). The organic matter (OM) and N analyses were conducted according to AOAC [29] methods 923.3 and 992.15, respectively, by ashing at 550 °C for 6 h for OM and using the Dumas method for N. The Crude Protein (CP) was estimated by multiplying N by 6.25. Analyses of the N content of fresh urine samples were performed using also the Dumas method. The cell wall components (NDF, ADF and acid detergent lignin (ADL)) in the diets and faeces were determined using a sequential procedure ([30], AOAC methods 200.04 and 973.18, respectively, for NDF and ADF + ADL, 15). Condensed tannins were extracted in an ultrasonic bath with a 70% (vol/vol) aqueous acetone solution [31] and isolated with Sephadex LH-20 (Sigma-Aldrich, St Louis, MO, USA). The CT content was determined using the vanillin–H_2_SO_4_ method described by Laurent [32]. A 70% (vol/vol) H_2_SO_4_ solution containing 1% vanillin (wt/vol) was added to the methanolic extract of the plant and the absorbance measured at 500 nm. The concentrations were determined using standards of tannin extracts from *Leucaena*.

### Statistical analyses

Intake, total tract digestibility, ADG, eosinophil numbers, PCV, and FEC were analysed in a randomised design using the mixed procedure of SAS [33]. Only the raw means of PCVs and FECs are reported in the tables. However, the PCVs and FECs were log-transformed (log (PCV + 1), log (FEC + 1)) before analysis. The analyses were performed taking into account treatment (n = 07), the week relative to the date of the artificial infection (n = 11) and the random effect of the animal (n = 55). Differences between means were tested using the pdiff option. Significance was declared at probability levels of ≤ 5%.

The model was:

$$ {Y}_{ijk}=\mu + Tri+{T}_j+{A}_k+{e}_{ijk} $$

where Y_*ij*_ is the explained variable, *µ* is the mean, *Tr*_*i*_ is the treatment fixed effect (I = 1–7), *T*_*j*_ is the week relative to the date of the artificial infection (*I* = 1–11), *A*_*k*_ is the random effect associated with the animal (*k* = 1–55), and e_*ijk*_ is the residual term.

The development of faecal eggs and the abomasum worm count were analysed in a randomised design using the General Linear Model procedure of SAS taking into account diet as a fixed effect. The pdiff option was used to test the differences between means.

## Abbreviations

ADF: Acid detergent fibre
ADG: Average daily growth
ADL: Acid detergent lignin
NCR: Conventional resources rich in protein
CT: Condensed tannins
CP: Crude protein
DM: Dry matter
ED: Effective degradability
FEC: Faecal egg counts
GIN: Gastrointestinal parasitic nematodes
IA: Kids infected with the susceptible strain and fed Diet 1
IL25: Kids infected with the susceptible strain and fed Diet 2
IL50: Kids infected with the susceptible strain and fed Diet 3
IL: Kids infected with the susceptible strain and fed Diet 4
NDF: Neutral detergent fibre
PCV: Blood packed cell volume
Ttd: Total tract digestion
UA: Uninfected kids fed Diet 1
UL: Uninfected kids fed Diet 4

## Availability of data and materials

Data supporting the conclusions of this article are included within the article. Raw data are available upon request from the corresponding author.
